# Sol-Gel Processing of MgF_2_ Antireflective Coatings

**DOI:** 10.3390/nano8050295

**Published:** 2018-05-02

**Authors:** Peer Löbmann

**Affiliations:** Fraunhofer-Institut für Silicatforschung, Neunerplatz 2, 97082 Würzburg, Germany; peer.loebmann@isc.fraunhofer.de; Tel.: +49-931-4100-404

**Keywords:** MgF_2_, antireflective coatings, sol-gel processing, mechanical stability, Ellipsometric Porosimetry

## Abstract

There are different approaches for the preparation of porous antireflective λ/4 MgF_2_ films from liquid precursors. Among these, the non-aqueous fluorolytic synthesis of precursor solutions offers many advantages in terms of processing simplicity and scalability. In this paper, the structural features and optical performance of the resulting films are highlighted, and their specific interactions with different inorganic substrates are discussed. Due to their excellent abrasion resistance, coatings have a high potential for applications on glass. Using solvothermal treatment of precursor solutions, also the processing of thermally sensitive polymer substrates becomes feasible.

## 1. Introduction

Antireflective surfaces are highly important for many applications such as photovoltaic and solarthermal panels, architectural glazing, display technology, optical instrumentations, and ophthalmic lenses. This is especially true if radiation has to pass multiple surfaces as in insulating windows or through different optical components. For antireflective purposes, diverse strategies (for instance, multilayer interference assemblies), single λ/4 layers, or index gradient surfaces may be pursued [1,2].

Antireflective interference filters have to consist of films with different refractive indices that may be deposited by vacuum-based technologies such as physical vapor deposition (PVD) or chemical vapor deposition (CVD). These single materials will basically exhibit a dense microstructure. As filters with a satisfactory optical performance have to consist of an at least 3-fold stack, multiple coating runs must be performed.

In contrast to this, antireflective properties can be established by a single film that mediates between the optical properties of the solid surface and the adjacent atmosphere. As the optimum transmittance is observed at a film thickness equal to one quarter of the wavelength of the incident light, these systems are referred to as λ/4 layers. Reflectivity is suppressed by destructive interference [3]. As for common optical glasses, the required low refractive index physically cannot be established by any dense material; porous structures are necessary. Pores can be introduced to glasses and polymers by etching [4]; in this case, though, large-scale industrial processing with high homogeneity is challenging. Films with a distinct porosity, however, may easily be deposited by sol-gel processing. Details of this approach will be discussed below.

Finally, antireflective properties may also be established by a continuous gradient of the refractive index from the exterior medium to the dense substrate [5]. According to their natural antetype, such features are referred to as “moth-eye” structures. To circumvent optical scattering, the size of the respective features has to be significantly smaller than the wavelength of the incident radiation. The direct embossing of formable substrates or films requires suitable stamper tools and refined processing technology. Under these constraints, cost efficient processing of large surfaces is a demanding task.

All strategies for antireflective surfaces, namely, λ/4 layers, multiple interference films, and “moth-eye” structures, may be addressed by sol-gel processing, as recently has been reviewed [1]: Firstly, chemical precursor solutions of the required composition are synthesized. Here, both purely inorganic systems [6] and hybrid polymers [7] are accessible. Films from these liquid sols are deposited by, for example, dip-coating, doctor-blading, spin-coating, or spraying. The deposits then have to be consolidated by thermal treatment (inorganic systems) or UV-curing (hybrids). As the annealing temperature for inorganic films commonly exceeds 300 °C, the use of thermally labile polymer substrates is restricted.

Dense sol-gel films of, e.g., SiO_2_, TiO_2_, ZrO_2_, or Al_2_O_3_ can be used for the production of multilayer interference antireflective assemblies. Formable films of inorganic [8] or hybrid polymer [9] composition may be embossed and result in durable “moth-eye” structures after curing. The above systems might well be obtained by alternative techniques. Nevertheless, the large-area processing of porous films, as required for λ/4 antireflective coatings, is a unique feature of sol-gel processing. While for the production of dense films the frequent remain of residual porosity is a drawback, tailored pore structures can be deliberately designed by the use of liquid precursors. Such features are difficult to address using other methods.

It is clear that λ/4 films only require a single coating, and they may be applied to large areas in high quality under commercial conditions at moderate plant-specific costs. In terms of stability, however, the porous structure of the materials represents a serious limitation. This is especially true in harsh environments, and when mechanical cleaning conditions have to be applied [10]. In this context, the optical properties, i.e., the refractive index, of the inorganic backbone material become important. In the case of SiO_2_ (*n* = 1.5) a porosity of 50% is required for common glasses to establish an effective refractive index of *n* = 1.22 of the overall film. This high porosity is the presumed reason why porous SiO_2_ antireflective coatings up to now have only been commercialized for solar panels in which no mechanical cleaning procedures are expected.

In order to attain a higher structural stability of λ/4 films, their porosity has to be reduced. To maintain optimum optical performance, a backbone material with a refractive index below that of SiO_2_ (*n* = 1.5) has to be applied. With *n* = 1.38, MgF_2_ is the material of choice.

In this paper, different methods for the sol-gel processing of MgF_2_ precursor are discussed. It turns out that the non-aqueous fluorolytic synthesis offers distinct advantages; the iterative improvement of this approach using various precursor chemicals is reviewed. Then, typical film microstructures and optical performances on glass substrates are highlighted, and specific MgF_2_ film interactions with different surfaces are debated. As they are of crucial importance for any practical application, the chemical and mechanical stability of MgF_2_ films are considered. Finally, the most recent developments enabling antireflective coatings on organic polymers are presented.

## 2. Synthetic Approaches

There are various reports concerning the chemical synthesis of liquid MgF_2_ precursor solutions, many of them being qualified for the sol-gel deposition of porous antireflective λ/4 layers. It is most convenient to subdivide these approaches according to the respective fluorine source used.

### 2.1. Fluorine Salts

When NaF is combined with MgCl_2_ or Mg(OAc)_2_ as magnesium sources, MgF_2_ particles with different morphology can be synthesized depending on the pH [11]. As the products are precipitates and even the primary particle sizes mostly exceed 100 nm, though, the preparation of antireflective coating seems impractical. The combination of KF with MgCl_2_ in the presence of a micellar environment of amphiphilic triblock copolymers yields significantly smaller particles [12], but MgF_2_ film deposition was not reported.

MgF_2_ average particle diameters between 11 and 261 nm were established by the combination of ammonium fluoride (NH_4_F) with MgCl_2_ [13]; the use of polystyrene (PS) templates results in the formation of elaborated raspberry-like structures [14]. Unfortunately, the deposition of thin films based on this approach in not yet disclosed.

Large (~35 µm) grains of magnesium fluoride hydrate (MgF_2_ × *x*H_2_O) were dissolved in *n*-propanol; in the presence of hydrochloric acid, porous particles in the size range around 50 nm are formed under solvothermal conditions at 200 °C. Using such precursor solutions, antireflective coatings were deposited on solar glass, a significant improvement of cell performance is claimed [15].

### 2.2. Trifluoroacetic Acid (TFA)

Many authors utilize trifluoroacetic acid (CF_3_COOH, TFA) in their synthesis [16]. Here, it has to be noted that these solutions do not yet contain MgF_2_ rather than magnesium coordinated by TFA. Nevertheless, these solutions can be used for the sol-gel deposition of thin films, but the final MgF_2_ composition is only obtained through the thermal treatment of the dried film. Some oxofluorides may be expected as byproducts [17,18]. From a practical viewpoint, the formation of volatile components such as (CF_3_CO)_2_O, CF_3_COF, and COF_2_ during thermolysis can be considered disadvantageous [19].

Mg(OAc)_2_ × 4H_2_O is commonly applied as magnesium source in combination with TFA. The role of heating rates during the film consolidation progress has been investigated [20]. Polyvinylacetate (PVA) can be used to induce phase separation in the films; using this tailored porosity the optical performance of large-area coatings on glass was optimized [21], and the annealing temperature was set to 450 °C. In the presence of tetraethyl orthosilicate (Si(OEt)_4_, TEOS), quaternary Mg–F–Si–O films were obtained with polyethylene glycol acting as the porogen [22].

### 2.3. Aqueous Hydrofluoric Acid

In most reports using aqueous HF, hydrothermal treatment is applied for precursor formation. When magnesium ethoxide Mg(OEt)_2_ is heated with HF in a Teflon coated container at 150 or 250 °C, MgF_2_ particles with diameters of 10 and 60 nm are formed respectively [23]. Using dip-coatings, antireflective films with a high laser induced damage thresholds were obtained [24].

Mg(OAc)_2_ is more commonly used as magnesium source. In early reports, MgF_2_ films from autoclaved sols are used for the preparation of antireflective coatings [25]. Subsequently, this approach was combined with an SiO_2_ binder [26]; the additional application of a fluorinated hydrocarbon leads to hydrophobic surfaces. In these studies, the AR properties are not primarily pursued any more [27,28].

The influence of autoclave temperature on MgF_2_ particle size was investigated [29]. In [30], the formation of hollow aggregates is induced by solvothermal treatment of Mg(OAc)_2_ at 150 °C; their morphological evolution is described. Similar structures are obtained at 180 °C [31]; here, the specific role of HCl for the structural evolution is discussed. All precursors can be used for the deposition of antireflective coatings.

Solvothermal treatment of Mg(OAc)_2_ with HF at 240 °C results in the formation of rod-like MgF_2_ structures that are used in combination with SiO_2_ particles for the preparation of hydrophobic antireflective coatings [32].

The application of an ultrasonic horn for the direct MgF_2_ coating of artificial teeth and other substrates is a rather extraordinary technique [33,34,35]. Here, also Mg(OAc)_2_ × 4H_2_O is used in combination with aqueous HF.

### 2.4. Non-Aqueous Hydrofluoric Acid

As the above synthetic approaches contain water, gelation is a general issue. In addition, residual oxide species in the final sol-gel product cannot be ruled out. Such impurities generally may increase the refractive index of the solid backbone material, and thus the film refractive index becomes enlarged. This is also true if oxofluoride species are formed during the thermal decomposition of TFA-based precursors. If aqueous HF remains in the coating solutions due to incomplete reaction, this represents a considerable health risk that is undesirable for any industrial processing. If solvothermal processing is imperative, this may also impede a commercial scale-up.

Some of these problems can be solved by applying the non-aqueous fluorolytic synthesis of precursor material [36,37]. Even though the handling of highly concentrated alcoholic HF solutions is demanding, the reactions are complete, and the products are harmless and non-toxic. MgF_2_ precursor solutions can be prepared with sufficient concentrations and high pot-life without the imperative need for any solvothermal treatment. Film processing is possible at moderate temperatures without the extensive formation of volatile products. In the following Section 3, Section 4, Section 5 and Section 6, the progression of the fluorolytic synthesis of MgF_2_ precursor solutions to a semi-industrial level is outlined.

The initial fluorolytic synthesis of MgF_2_ solutions for the preparation of thin films was based on suspensions of Mg(OMe)_2_ in methanol [38]. It turned out, though, that it was difficult to establish a reliable commercial supply of this compound. Therefore, magnesium methoxide was synthesized by the in-situ dissolution of metallic Mg in MeOH [39]. Subsequent fluorination was carried out by adding water-free HF dissolved in methanol, yielding a turbid solution that cleared up within several days. Deagglomeration of the particles was monitored by measurements of the hydrodynamic size [40]. After sufficient ageing, porous MgF_2_ coatings with excellent optical properties could be prepared.

**Figure 1:**
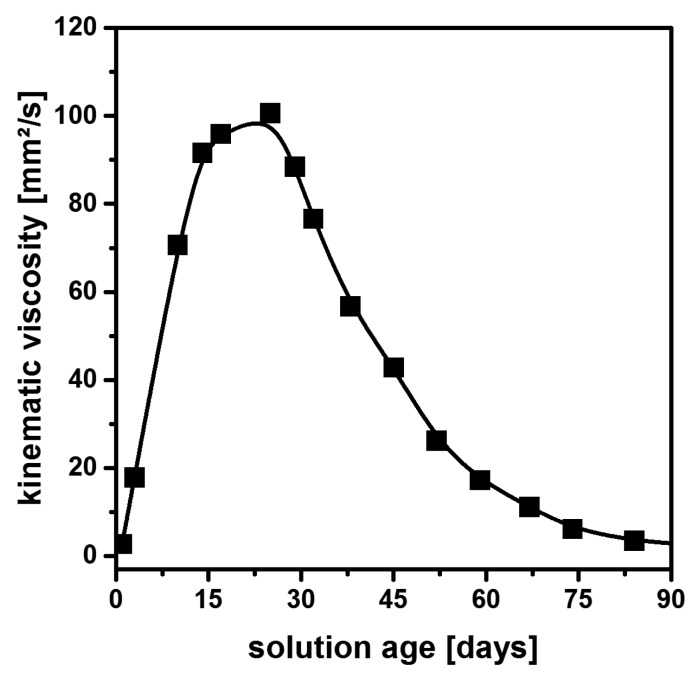
Viscosity of an MgF_2_ precursor solution prepared from the reaction of Mg(OMe)_2_ with anhydrous HF as a function of sol age. Based on [41].

Despite their initial turbidity, however, these solutions regularly exhibit unusual rheological behavior [41], as shown in Figure 1: The viscosity of the as-prepared material first increases until a maximum is reached in approximately 20 days. It then decreases again until the initial level is attained again after 90 days. As the film thickness in dip-coating experiments critically depends on the flow characteristics of the solution, this behavior is highly undesirable from the viewpoint of any industrial processing. In addition, the formation of hydrogen during the dissolution of metallic magnesium and the toxicity of methanol are extra drawbacks from a practical point of view.

**Figure 2:**
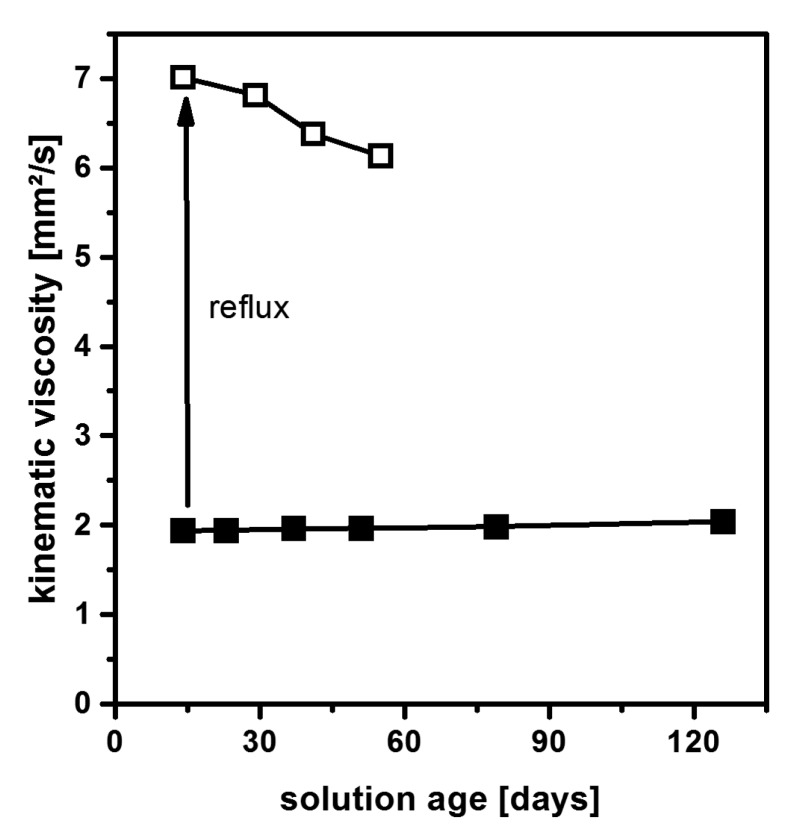
Viscosity of an MgF_2_ precursor solution prepared from the reaction of MgCl_2_ with anhydrous HF as a function of sol age. Data are given for the as-prepared sol (■) and samples that have been refluxed for 24 h (□). Reproduced with permission from [42]. Copyright The Royal Society of Chemistry, 2012.

Even though Mg(OEt)_2_ has a better commercial availability than Mg(OMe)_2_, this compound is neither soluble in ethanol nor in methanol. Therefore, MgCl_2_ was examined as a possible starting material [42]. The fluorolytic synthesis in ethanol readily yields MgF_2_ particles. While minor amounts of unreacted HF were initially detected on their surface, these traces disappear after ageing. In contrast to material prepared from Mg(OMe)_2_, the initial viscosity remains constant over a large period of time (Figure 2). Even when particle growth [42] is induced by boiling of the precursor solution, the viscosity is only gradually increased and remains in a range suitable for commercial production.

Thin films with excellent antireflective properties and a remarkable abrasion resistance were prepared from these precursors. During the synthesis, however, HCl is produced, making the solutions highly acidic. As this gas is liberated during film drying and thermal annealing, industrial production equipment will be corroded. Therefore, the search for alternative starting materials was continued.

**Figure 3:**
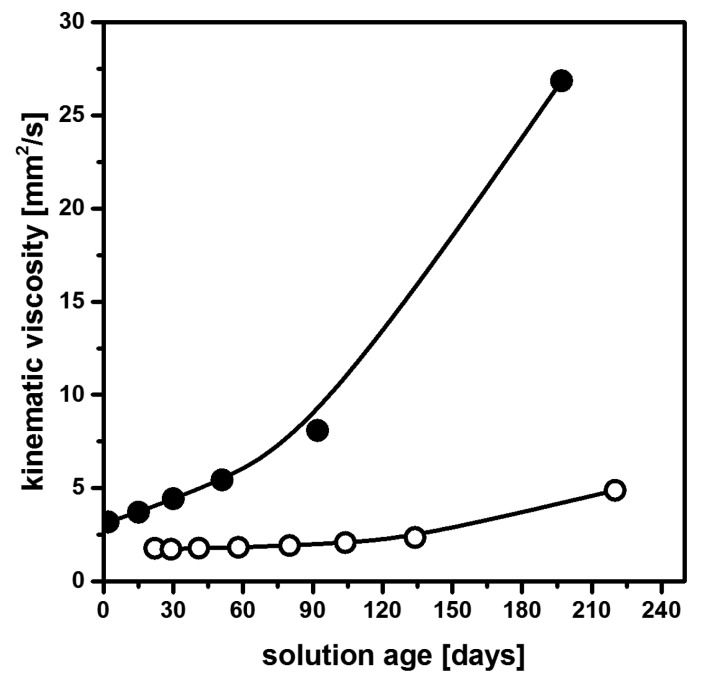
Viscosity of MgF_2_ precursor solutions prepared from the reaction of Mg(OAc)_2_ × 4H_2_O (●) and dried Mg(OAc)_2_ (○) with anhydrous HF as a function of sol age. Reproduced with permission from [43]. Copyright The Royal Society of Chemistry, 2015.

Mg(OAc)_2_ × 4H_2_O is a compound commonly used in the processing of MgF_2_ using aqueous HF (see Section 2.3). Clear solutions can also be obtained in water-free fluorolysis [43], but their viscosity constantly increased during aging, as can be seen in Figure 3. Water adsorbed to the MgF_2_ particles is believed to result in surface –OH groups that then undergo condensation reactions. The consequential particle aggregation then leads to the observed increase of viscosity and subsequent gelation [43]. Unfortunately, Mg(OAc)_2_ × 4H_2_O dehydrated at 210 °C in air or commercial anhydrous Mg(OAc)_2_ turned out to be only partially soluble in ethanol. Soluble Mg(OAc)_2_, however, can be obtained by gentle drying at 100 °C in vacuum. MgF_2_ coating solutions based on these precursors exhibit a small viscosity that only moderately raises upon aging (Figure 3).

Even though water is excluded from the synthesis procedure, acetic acid is generated by the fluorolysis reaction of Mg(OAc)_2_. Esterification of this CH_3_–COOH with the alcoholic solvent, however, results in the formation of H_2_O and a viscosity rise in the long term.

The initial turbidity of many sols synthesized with anhydrous hydrofluoric acid is attributed to minor amounts of unreacted HF on the particle surfaces. These traces thus can result in temporary formation of hydrogen bonds and aggregation. The sols then clear up when the fluorolysis comes to completion during aging. This process is accelerated by the addition of small quantities of, e.g., Al(O^i^Pr)_3_ that quickly binds the residual HF [43]. As a side-effect, some water generated by ester formation may be removed by the hydrolysis of the metal alkoxide. This concept, however, is only bearing in a limited range: Larger amounts of oxides from such side-reactions will alter the composition of the system and thus increase its refractive index. The long-term stability of the Mg(OAc)_2_-based solutions thus is an inherent unsolved problem. Additionally, the gentle dehydratization of Mg(OAc)_2_ × 4H_2_O under vacuum is a laborious time-consuming process; therefore, additionally, this synthesis route only has a narrow commercial perspective.

As the fluorolytic synthesis of MgF_2_ from Mg, Mg(OMe)_2_, MgCl_2_, and Mg(OAc)_2_ sets free the undesirable by-products H_2_, MeOH, HCl, and CH_3_–COOH, Mg(OEt)_2_ would be the preferential precursor that was only eliminated due to its insufficient solubility. Even if a suspension of Mg(OEt)_2_ was employed, anhydrous HF would only react to form an insoluble MgF_2_ protection layer on its surface, which impedes complete transformation to colloidal MgF_2_. Therefore, a reaction was searched to convert Mg(OEt)_2_ into a soluble reactive intermediate [44]

**Figure 4:**
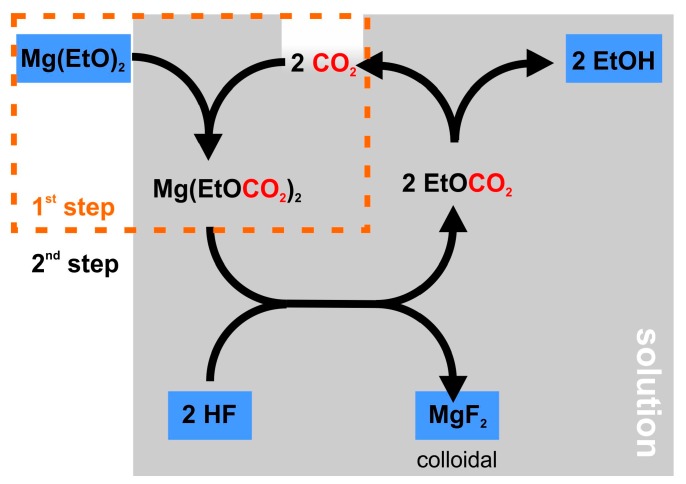
Reaction scheme of the fluorolytic synthesis of colloidal MgF_2_ from Mg(OEt)_2_ using CO_2_ to form a soluble intermediate magnesiumethyl-carbonate species Mg(EtOCO_2_)_2_ [44].

It was found that the weak Lewis acid CO_2_ reacts with Mg(OEt)_2_, forming soluble magnesiumdiethylcarbonate Mg(EtOCO_2_)_2_. With HF, this compound readily forms colloidal MgF_2_ and ethylcarbonate. The later product immediately decomposes into EtOH and CO_2_. The overall reaction scheme is visualized in Figure 4. From this viewgraph, it appears as if only catalytical amounts of carbon dioxide are required, as it is not stoichiometrically consumed. It has to be noted, though, that the formation of Mg(EtOCO_2_)_2_ has to be completed in a first reaction step before the addition of HF. Otherwise, as stated above, the surface of Mg(OEt)_2_ particles in suspension will be passivated by an insoluble MgF_2_ barrier layer [44].

In order to establish a more simple one-step synthesis, a similar approach using HCl and MgCl_2_ as intermediate species was developed that steadily dissolves magnesium from the surface of dispersed Mg(OEt)_2_ [44].

In Section 2, several strategies for the synthesis of precursors leading to MgF_2_ films have been reviewed. It appears as if the non-aqueous route (Section 2.4) offers some distinct advantages in terms of toxicity, scalability, or purity of the final product. It turned out that layers originating from this general approach may, for example, gradually differ in their respective microstructure depending on the specific synthesis conditions and Mg precursor used. Nevertheless, many properties such as optical performance, behavior through thermal processing, interaction with the substrate, and mechanical stability can be considered as universal features of these films. The subsequent Section 3, Section 4, Section 5 and Section 6 therefore are focused on the review of such general characteristics.

## 3. Film Microstructure and Optical Performance

**Figure 5:**
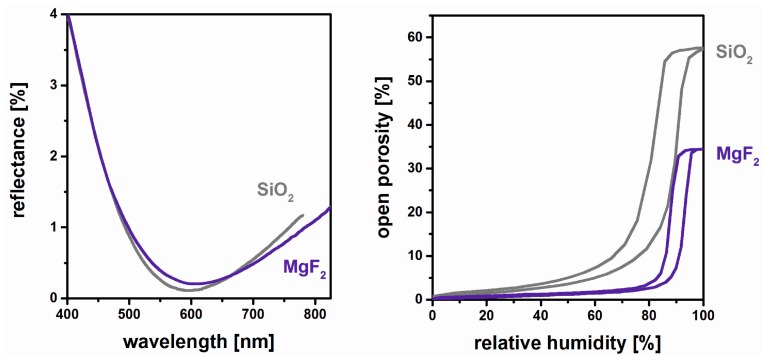
Reflectance (**left**) and open porosity as measured by Ellipsometric Porosimetry (**right**) of porous SiO_2_ and MgF_2_ coatings. Based on [41].

It was already mentioned that for MgF_2_ films such as λ/4 antireflective coatings, only a reduced porosity is required compared to their SiO_2_ counterparts. This can be expected to be highly advantageous with regard to mechanical durability. In Figure 5, the transmittance of such exemplary films and their respective open porosity as measured by Ellipsometric Porosimetry (EP) [45] are compiled [41]. As both systems show comparable and excellent antireflective properties, this performance is achieved with 34% porosity by MgF_2_, whereas SiO_2_ requires 55% porosity.

**Figure 6:**
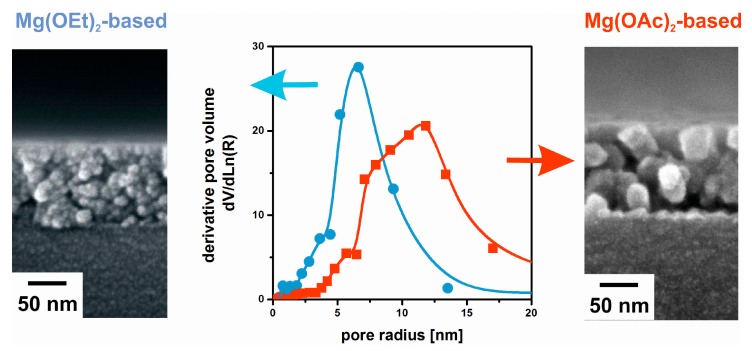
SEM cross-sectional images of two MgF_2_ thin films based on Mg(OEt)_2_ (**left**) and Mg(OAc)_2_ (**right**). The pore radius distributions as determined by Ellipsometric Porosimetry are given in the **middle**. Based on [41].

If the refractive index of the backbone material is the same, the optical performance is determined solely by porosity and thickness rather than by the microstructure of the films. MgF_2_ coating solutions were prepared by the non-aqueous fluorolysis of Mg(OMe)_2_ and Mg(OAc)_2_ [41]. The sols using Mg(OMe)_2_ result in a finer granular structure than those from Mg(OAc)_2_, as can be seen in the SEM (Scanning Electron Microscopy) images in Figure 6. These features are also confirmed by their respective pore radius distribution. Nevertheless, despite the significant morphological differences the antireflective properties turn out to be virtually the same due to identical porosity.

During sol-gel processing of inorganic films, thermal annealing is commonly applied to decompose residual organics, consolidate their microstructure, induce crystallization, and thus establish stable final properties. Therefore, it is important to monitor the MgF_2_ coatings throughout this treatment.

**Figure 7:**
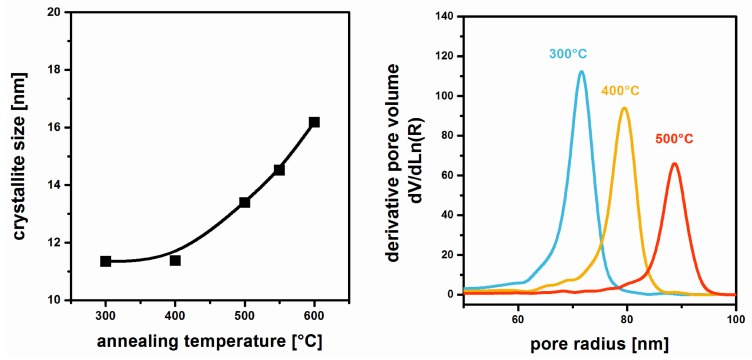
Crystallite size derived from XRD data (**left**) and pore radius distributions as determined by Ellipsometric Porosimetry (**right**) of MgF_2_ films annealed at different temperatures. Reproduced with permission from [42]. Copyright The Royal Society of Chemistry, 2012.

MgF_2_ thin films based on MgCl_2_ were deposited on borosilicate glass [42]. Crystalline phases can be detected by X-ray diffraction (XRD) for annealing temperatures exceeding 300 °C; the respective grain sizes as determined by the Scherrer-equation are shown in Figure 7. It can be seen that from 400 to 600 °C, the crystallites steadily grow from approximately 11 to 16 nm. This rise goes along with a significant increase of the pore radius as determined by Ellipsometric Porosimetry (Figure 7).

**Figure 8:**
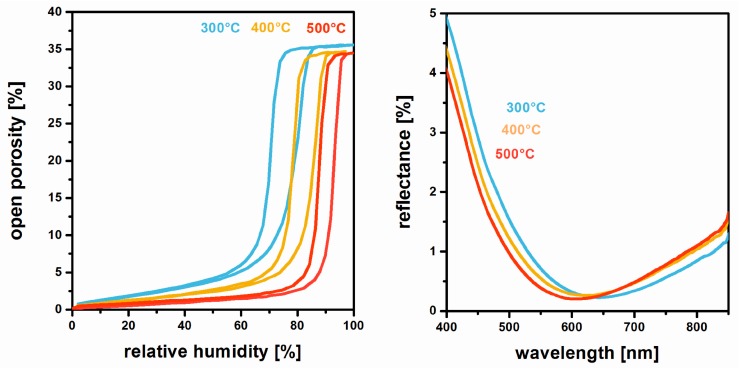
Water vapor sorption isotherms (EP) (**left**) and reflectance (**right**) of MgF_2_ films annealed at different temperatures. Reproduced with permission from [42]. Copyright The Royal Society of Chemistry, 2012.

These microstructural changes, however, only go along with a minor decrease in film porosity (Figure 8) that stays in the range between 33% and 35%. As a consequence, good optical performance is maintained [42]. This benign thermal behavior is representative of antireflective MgF_2_ prepared by the non-aqueous fluorolytic processing of different Mg source materials.

Structural changes induced by the film interaction with the substrate in multiple coating will be discussed in the following Section 4.

## 4. MgF_2_ Film Interaction with Substrate

Sol-gel films of a given thickness of, e.g., 100 nm can be applied by a single dip-coating experiment. Alternatively, the same width may be achieved by multiple deposition procedures with smaller layer thicknesses. If, in our example, five steps are performed, a respective single layer thickness of 20 nm must be established. As this thickness decreases, the role of the underlying substrate in relation to the film “bulk” volume gains importance as nucleation site for material densification and crystallization. Owing to the reduction of the single layer thickness, the film density is reduced, and even columnar microstructures without residual porosity can be obtained. This phenomenon has been described in detail for sol-gel derived titania films [46]. It has to be noted that the solid amorphous glass surface below the first deposit plays the same general role as the underlying “homoepitactic” films of the respective film composition of the subsequent coatings. However, as any crystalline bottom material has an improved effect on crystallization, the transition of a granular to a columnar microstructure can be observed from the bottom to the surface of such multilayers [46].

**Figure 9:**
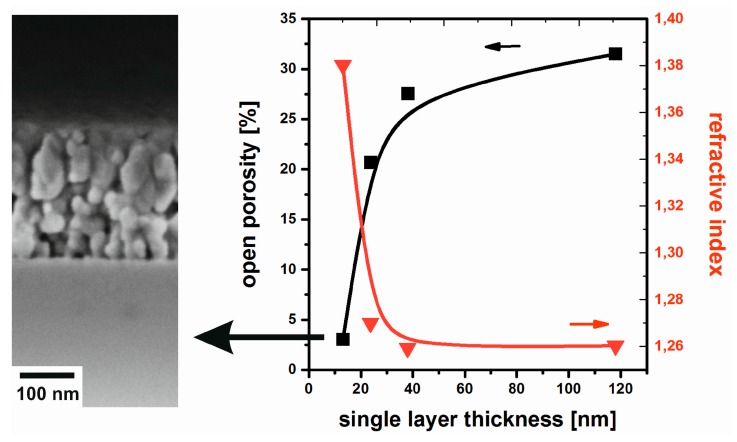
Open porosity and refractive index of MgF_2_ films with different single layer thickness. The inset shows the SEM image of a 15-fold coating. Based on [48].

The MgF_2_ films show a similar behavior than oxide-based materials; the above general observations were previously reported for TiO_2_ [46] and ZnO [47]. As displayed in Figure 9, the porosity is reduced from the ~30% typically observed for single layers to values below 5% for 15-fold coatings [48]. Compared to the examples in Figure 6, also the microstructure is altered: The SEM image in Figure 9 reveals the transition from the more granular morphology near the glass substrate to columnar features closer to the outer film surface. The refractive index of these samples approximates the theoretical value of dense MgF_2_.

Due to the amorphous structure of glass, the above initial interaction with MgF_2_ solely depends on its dense nature. If MgF_2_ is deposited on crystalline TiO_2_ films, however, a distinct heteroepitactic interaction between the two systems can be detected [48]. The reason for this behavior lies in the same tetragonal crystal structure of both phases.

**Figure 10:**
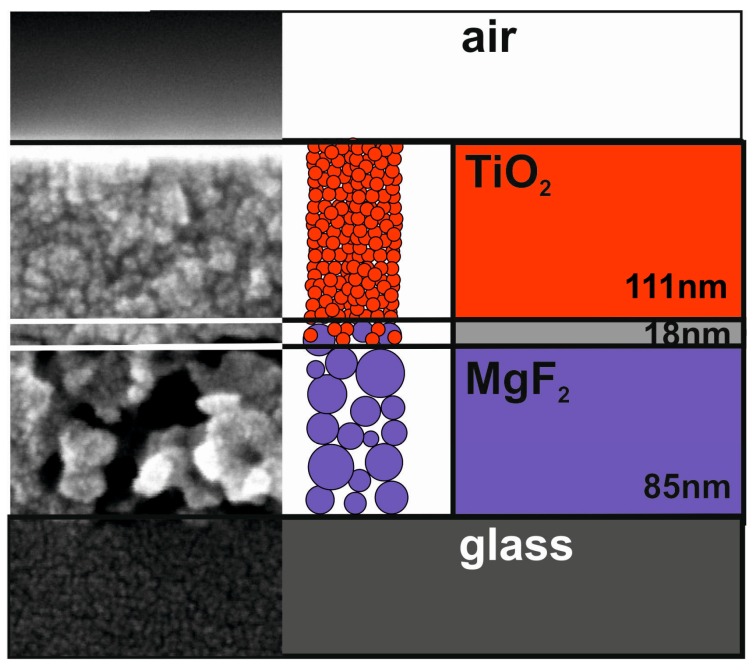
SEM image along with schematic representation of sol-derived TiO_2_ film on top of porous MgF_2_ layer. Based on [50].

The findings regarding MgF_2_ film densification and specific MgF_2_–TiO_2_ interactions are certainly of academic interest. When high-index TiO_2_ is combined with MgF_2_ in interference filters [41,49], however, for MgF_2_ a porous microstructure with a low refractive index is required. In this context the morphological interplay is of higher importance. It could be shown that titania particles from coating solutions only infiltrate underlying porous MgF_2_ films to a small extent. In Figure 10, the joining of the SEM image of such a bilayer assembly with its schematic representation is given. Film thickness parameters of TiO_2_, MgF_2_, and the mixed interlayer were derived from Ellipsometer measurements [50].

Despite any structural or crystallographic interactions, the substrate may react with the film material. For samples prepared on borosilicate glass, it was observed that the MgF_2_ reacts with silica according to

2 MgF_2_ + SiO_2_ → 2 MgO + SiF_4_ (g)

**Figure 11:**
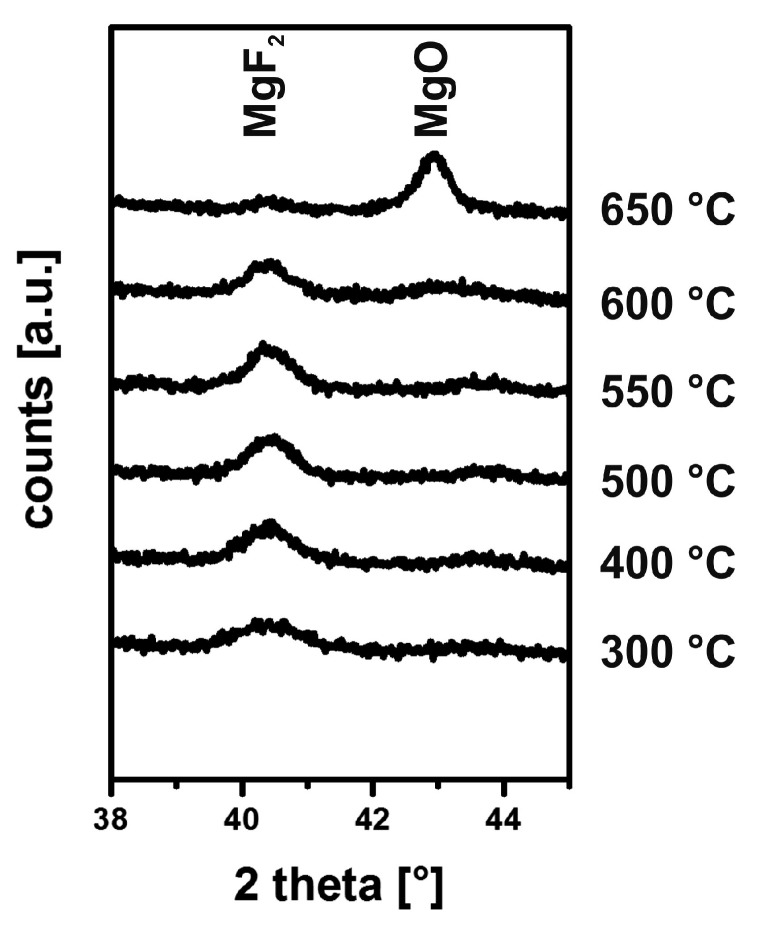
XRD pattern of MgF_2_ films on borosilicate glass. Reproduced with permission from [42]. Copyright The Royal Society of Chemistry, 2012.

The related XRD patterns are shown in Figure 11. After treatment, a 600 °C slight decrease of the MgF_2_ reflex may be adumbrated along with the occurrence of a broad hump around 43 °C. At 650 °C, this weak signal increased to a clear reflex of MgO, whereas the MgF_2_ almost vanished [42]. Corresponding to the above equation, fluorine is quantitatively removed from the system as volatile SiF_4_. Fortunately, this conversion only takes place above the glass transition temperature of the most common substrates and therefore is not relevant to any manufacturing process.

**Figure 12:**
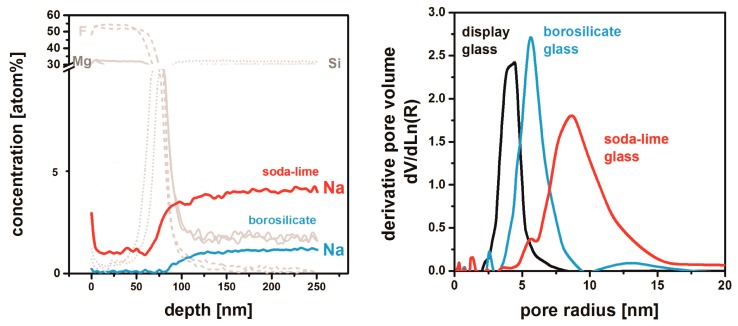
XPS depth profile of MgF_2_ films deposited on soda-lime and borosilicate glass (**left**) and pore radius distributions of samples on different glasses as measured by EP (**right**). All films have been thermally treated at 500 °C. Based on [51].

The glass substrate may interfere with the MgF_2_ material in a more subtle way by the elution of metal ions: MgF_2_ thin films were prepared on soda-lime, borosilicate, and Na-free display glass [51]. XPS (X-ray Photoelctron Spectroscopy) depth profiling (Figure 12) reveals that significant amounts of sodium diffuse into the MgF_2_ material presumably during thermal annealing. This effect is stronger for Na-rich soda-lime glass than for borosilicate substrates; it is self-evident that no sodium contamination takes place from Na-free display glass.

The sodium originating from the substrates has a pronounced impact on the MgF_2_ microstructure that is generated upon thermal treatment. Whereas films on soda-lime glass exhibit a maximum of their pore radius distribution around 9 nm, the pores of MgF_2_ deposited on borosilicate glass are significantly smaller. Following this logical line of argument, the tiniest pores are found on Na-free display glass. As these structural effects are also reflected in different levels of film stability, these findings are of high practical importance and will be discussed in the next Section 5.

## 5. Chemical and Mechanical Film Stability

**Figure 13:**
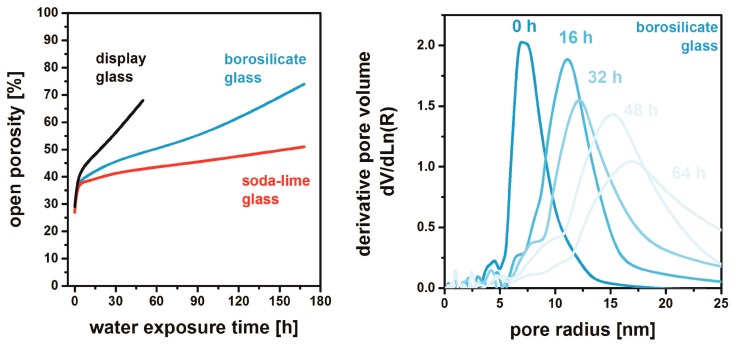
Open porosity of MgF_2_ thin films deposited on different substrates as a function of exposure time to water (**left**) and the respective pore radius distributions of the samples from borosilicate glass (**right**). Based on [50].

MgF_2_ thin films show a certain solubility against exposure to liquid water resulting in delamination after prolonged contact [51]. The speed of degradation depends on the nature of the glass substrate used respectively. In Figure 13, the open porosity of different films is monitored during water exposure. For all samples, swift increase in porosity is observed within the first hours; from then on, the process is slowed to different extents. MgF_2_ deposited on display glass shows the highest rate of dissolution: the films delaminate after 45 h. For borosilicate glasses, this degradation is significantly slower, whereas the lowest rate is observed on soda-lime glass. The leaching of the films proceeds by a continuous growth of the pore size as exemplified for MgF_2_ on borosilicate glass in Figure 13.

As discussed in Section 4, sodium from the glass substrates seemingly coarsens the pore structure and promotes grain growth. The resulting microstructure (lower specific surface) obviously affects the dissolution characteristics. An additional favorable influence of a reduced solubility of MgF_2_ by incorporation of Na into the lattice may be effective, but cannot be separated from the structural effect [51].

It was shown that doping of MgF_2_ precursor solutions with Na has a similar consequence, and the film material can be stabilized against dissolution by this means, irrespective of the substrate used [51].

**Figure 14:**
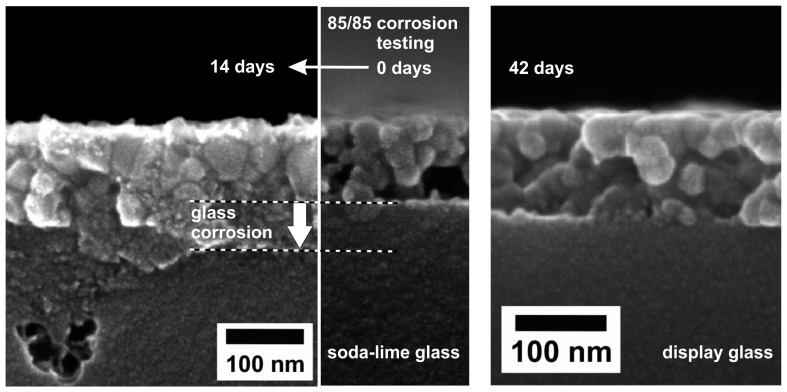
SEM cross-sectional views of MgF_2_ thin films on different glass. Based on [41].

In order to investigate the durability of films under atmospheric conditions, harsh conditions such as the “85/85 testing” were applied. In this steady-state, temperature humidity life test coated samples are exposed to 85% relative humidity at 85 °C in a climate chamber [41]. In Figure 14, the results of such experiments are given. Samples prepared on soda-lime glass visually show severe deteriorations after 14 days. When compared to the initial state, the MgF_2_ film is located on top of a distinct region of corroded soda-lime glass, so that upon first examination the film thickness seemingly increased. Display glass, however, has a significantly higher corrosion resistance. Therefore, MgF_2_ films deposited on such substrates basically appear unaltered even after 42 days of 85/85 testing. In case of these specific testing conditions, the substrate stability apparently is the limiting factor—the MgF_2_ film material itself shows an excellent persistence. In comparison to the results formerly discussed (Figure 12), it has to be noted that the impact of liquid water is much more critical for porous MgF_2_ films than the contact to humid atmosphere at elevated temperatures.

It is noteworthy that regarding solubility in liquid water and damp heat stability, the different glasses have adverse effects. On the one side, the solid film backbone is stabilized by sodium from soda-lime glass; on the other side, this substrate is more prone to corrosion itself. Whereas MgF_2_ on display glass shows highest solubility, this composition has a higher stability as a substrate.

**Figure 15:**
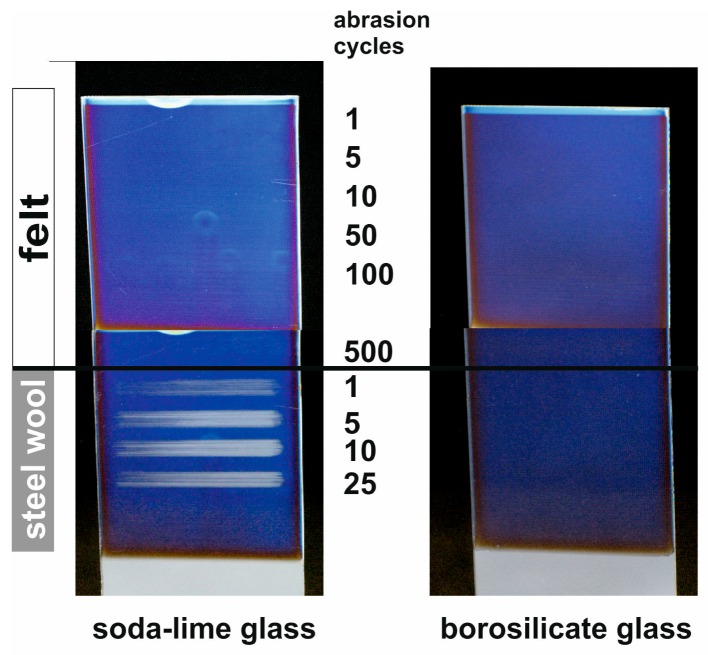
Photographs of MgF_2_ films on soda-lime glass (**left**) and borosilicate glass (**right**) substrates (width 10 cm) after different numbers of Crockmeter testing cycles with felt and steel wool as abrasive medium. Reproduced with permission from [42]. Copyright The Royal Society of Chemistry, 2012.

Besides chemical permanence, the mechanical film properties are of crucial importance for any application. As already revealed in Section 3, MgF_2_ thin films require a significantly lower porosity for a similar antireflective performance (Figure 5). For MgF_2_ films prepared from MgCl_2_ precursor, Crockmeter testing was applied to investigate their abrasion resistance [42]. In the course of this method, stamper are applied under constant load in a translator motion to the film surface. Normally, felt is used as an abrasive medium. On the MgF_2_ films under investigation, however, no damage was induced by such stampers even after 500 cycles. Therefore, steel wool was applied as a tougher abrasive medium. In Figure 15, the results for MgF_2_ films prepared on soda-lime and borosilicate glass are displayed. On both substrates, the coatings remain unaffected even after 500 cycles using felt. The surface of MgF_2_ on soda-lime glass is damaged by the first load of steel-wool, 25 cycles cause extensive abrasion. In contrast to that, no significant marks can be detected on borosilicate glass.

It was discussed above that Na effusion from soda-lime glass resulted in increased grain growth compared to MgF_2_ deposited on borosilicate substrates. One may expect that this is more likely to result in higher mechanical stability. It has to be considered, though, that Crockmeter testing rather provides information about the film adhesion to the substrate than about the mechanical strength of the film backbone. In this context, the respective bonding to the glass surface may be the determinant factor. Obviously, there is a wide range for fundamental research to elucidate this background.

Regarding practical applications of MgF_2_ antireflective coatings, however, it is important to compare the performance of different commercial products. Three sol-gel-based products were compared to MgF_2_ thin films [52]. The system commercialized by Prinz Optics is a 3-layer antireflective stack, whereas products provided by the company DSM (Heerlen, The Netherlands) and Centrosolar are λ/4 single layer coatings based on porous SiO_2_. For all specimens, a peak transmittance exceeding 97.5% could be observed.

**Figure 16:**
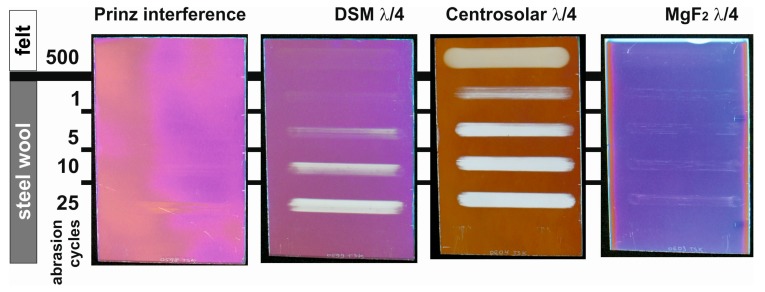
Photographs of commercial sol-gel-based antireflective systems after Crockmeter testing in comparison to MgF_2_ λ/4 films. Based on [52].

In Figure 16, the results of Crockmeter testing procedures are compared. As one would expect from the dense microstructure of the interference filter by Prinz Optics, no extensive damage is observed even after 25 cycles using steel wool as abrasive. The DSM system consists of mesoporous SiO_2_ that was created by the thermolysis of organic templates. These films show first scratches after 5 loadings with steel wool; after 25 cycles the film is mostly removed. The microporous SiO_2_ coatings manufactured by Centrosolar are already completely detached by 500 cycles using felt. In contrast to that, the MgF_2_ films under investigation only show minute marks after 25 loadings with steel wool. Hence, the abrasion resistance of porous MgF_2_ is comparable to that of the dense interference stack by Prinz Optics offering a high potential for future commercialization.

## 6. Coating of Polymer Substrates

The coatings discussed in Section 2, Section 3, Section 4 and Section 5 originate from as-prepared, non-aqueous fluorolytic MgF_2_ solutions. For all samples, glass substrates had to be used, since a thermal treatment of at least 300 °C is required to remove residual organics and to provide sufficient antireflective properties. These conditions rule out transparent polymers.

**Figure 17:**
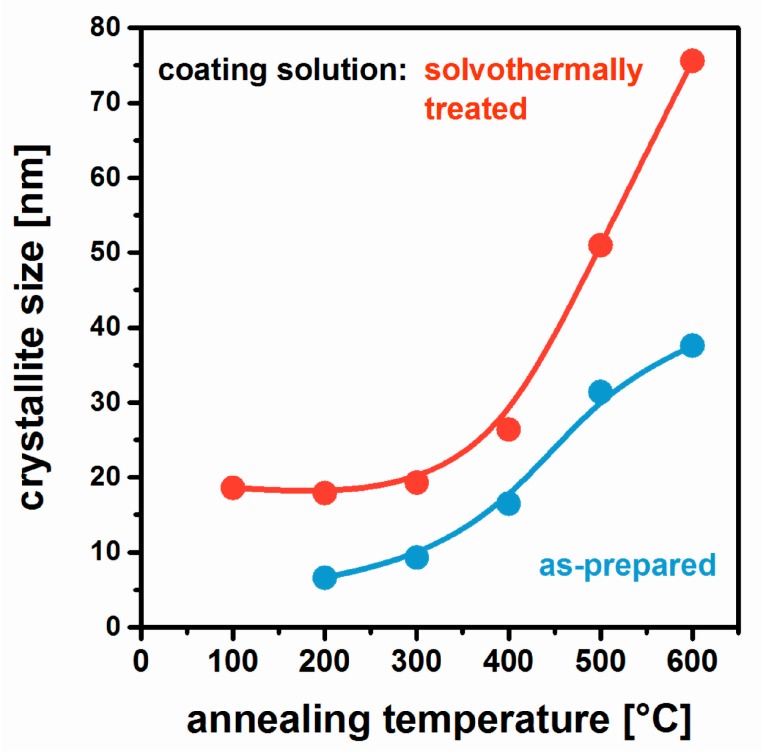
MgF_2_ crystallite size as determined by XRD of films using as-prepared and solvothermally treated coating solutions as a function of annealing temperature. Based on [53].

In order to circumvent these restrictions, MgF_2_ coating solutions from non-aqueous synthesis (see Section 2.4) were solvothermally treated at 160 °C [53]. The resulting clear products could be used for further film deposition without any additional filtration. In a first step, glass substrates were coated and thermally annealed in order to investigate their influence on the crystallization process. In Figure 17, the respective crystal sizes are summarized. From as-prepared MgF_2_ solutions, first diffraction patterns analyzable by the Scherrer-equation are obtained at 200 °C; up to 600 °C, the MgF_2_ grains grow from 6.6 to 38 nm. For films originating from solvothermally treated sols, crystallites of 19 nm are already observed at 100 °C. They remain basically unchanged up to 300 °C; from then on, an increase to 76 nm at 600 °C takes place. In summary, it can be concluded that the solvothermal treatment induces MgF_2_ crystallization already in the liquid state, and larger grain sizes are maintained throughout thermal processing in comparison to as-prepared coating solutions.

**Figure 18:**
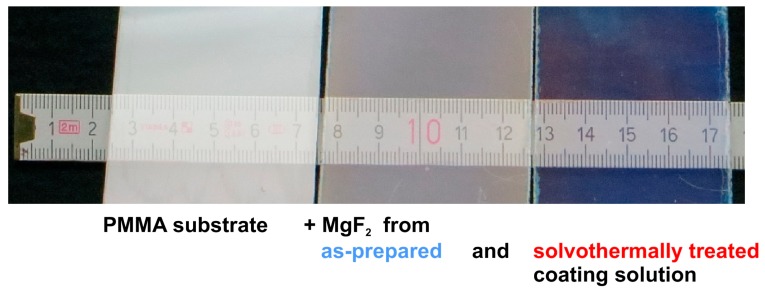
Photographs of bare PMMA substrate (**left**), PMMA coated with as-prepared MgF_2_ coating solution (**middle**), and coating solution that had undergone solvothermal treatment (**right**). Based on [53].

Both coating solutions show good wetting behavior to PMMA (Poly(methyl methacrylate)) surfaces. In Figure 18, bare PMMA substrates are compared to samples coated by as-prepared sols and solvothermally treated MgF_2_ coating solutions. A drying step at just 80 °C was performed. Both films appear homogeneous and crack-free, but only the samples obtained using the modified precursor exhibit the blueish coloration typical of antireflective coatings.

**Figure 19:**
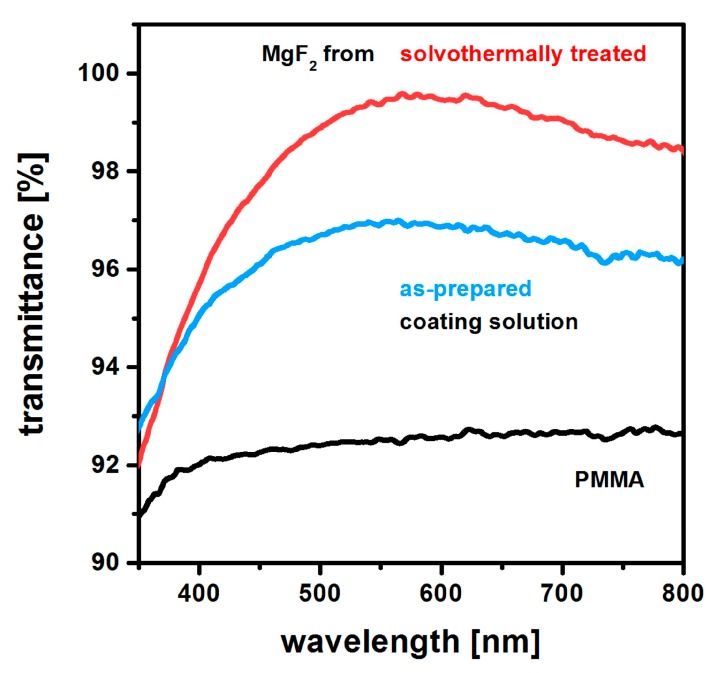
Transmittance of MgF_2_ films from solvothermally treated and as-prepared coating solutions. For comparison data from an uncoated, PMMA substrate is given. Based on [53].

The visual findings of Figure 18 were quantified by UV-Vis spectroscopy. As can be seen from Figure 19, the films from as-prepared sols only provide a limited level of antireflective properties. EP investigations [53] reveal that such layers only have an open porosity of 28%. In contrast to that for films from solvothermally modified solutions, a porosity of 33% is measured. For these systems, a peak transparency exceeding 99% is demonstrated by Figure 19. It has to be noted, however, that the optical performance is determined by both the film porosity and the refractive index of the solid backbone. In this case, these two factors cannot be clearly discriminated. On the basis of Figure 17, it can be assumed, though, that the solid skeleton from the solvothermally treated sol is closer to the theoretical level of MgF_2_ than that using the as-prepared precursor.

In summary, porous MgF_2_ offers vast potential for antireflective coatings on organic polymers. In order to achieve the excellent abrasion resistance as observed on glass (Section 5), future research has to be undertaken.

## 7. Conclusions and Outlook

The non-aquous fluorlytic synthesis for MgF_2_ coating solutions offers many advantages over alternative routes relating to the toxicity, scalability, and purity of the final product. The use of Mg, Mg(OMe)_2_, MgCl_2_, and Mg(OAc)_2_ as magnesium source, however, resulted in problems in terms of solution stability, availability of the source materials, and undesirable by-products. The insolubility of Mg(OEt)_2_ could be overcome by the application of CO_2_ or HCl as intermediate species; all problems in the context of the other Mg precursors were eliminated.

MgF_2_ thin films on glass show a remarkable stability in a broad range of treatment temperatures. Despite particle- and pore-growth, the porosity is maintained up to 600 °C, guaranteeing excellent antireflective properties. The structural interaction with substrate surfaces and underlying films is well understood. Even though MgF_2_ may react with silica from glass to MgO at 650 °C, this high temperature is irrelevant to any practical hardening conditions.

MgF_2_ films were found to take up sodium from alkali containing glass compositions. As this process does stabilize the material against dissolution in liquid H_2_O, it is generally beneficial. Similar protection can also be established by doping the precursor solution with Na ions. Regarding corrosiveness of moist atmosphere at 85 °C, MgF_2_ turns out to be highly stable even compared to soda lime substrates. Antireflective MgF_2_ coatings can withstand Crockmeter testing, even when steel wool is used as abrasive. In this respect, commercial SiO_2_-based λ/4 films are clearly outperformed.

Through the use of solvothermally-treated MgF_2_ solutions, antireflective coatings can also be prepared on thermally unstable polymer substrates with peak transmittances exceeding 99%.

In summary, the sol-gel preparation of MgF_2_ films from non-aqueous fluorolytic synthesis offers bright prospects for the commercialization of next-generation λ/4 antireflective coatings on glasses and polymer surfaces.
